# Myoglobin Interaction with Lactate Rapidly Releases Oxygen: Studies on Binding Thermodynamics, Spectroscopy, and Oxygen Kinetics

**DOI:** 10.3390/ijms23094747

**Published:** 2022-04-26

**Authors:** Kiran Kumar Adepu, Dipendra Bhandari, Andriy Anishkin, Sean H. Adams, Sree V. Chintapalli

**Affiliations:** 1 Arkansas Children’s Nutrition Center, Little Rock, AR 72202, USA; bhandarid@archildrens.org; 2 Department of Pediatrics, University of Arkansas for Medical Sciences, Little Rock, AR 72202, USA; 3 Department of Biology, University of Maryland, College Park, MD 20742, USA; anisan@gmail.com; 4 Department of Surgery, School of Medicine, University of California, Davis, CA 95616, USA; shadams@ucdavis.edu; 5 Center for Alimentary and Metabolic Science, University of California, Davis, CA 95616, USA

**Keywords:** binding, lactate, lactic acidosis, myoglobin, oxygen release

## Abstract

Myoglobin (Mb)-mediated oxygen (O_2_) delivery and dissolved O_2_ in the cytosol are two major sources that support oxidative phosphorylation. During intense exercise, lactate (LAC) production is elevated in skeletal muscles as a consequence of insufficient intracellular O_2_ supply. The latter results in diminished mitochondrial oxidative metabolism and an increased reliance on nonoxidative pathways to generate ATP. Whether or not metabolites from these pathways impact Mb-O_2_ associations remains to be established. In the present study, we employed isothermal titration calorimetry, O_2_ kinetic studies, and UV-Vis spectroscopy to evaluate the LAC affinity toward Mb (oxy- and deoxy-Mb) and the effect of LAC on O_2_ release from oxy-Mb in varying pH conditions (pH 6.0–7.0). Our results show that LAC avidly binds to both oxy- and deoxy-Mb (only at acidic pH for the latter). Similarly, in the presence of LAC, increased release of O_2_ from oxy-Mb was detected. This suggests that with LAC binding to Mb, the structural conformation of the protein (near the heme center) might be altered, which concomitantly triggers the release of O_2_. Taken together, these novel findings support a mechanism where LAC acts as a regulator of O_2_ management in Mb-rich tissues and/or influences the putative signaling roles for oxy- and deoxy-Mb, especially under conditions of LAC accumulation and lactic acidosis.

## 1. Introduction

Lactate (LAC), once solely considered a product of anaerobic glycolysis, is now recognized to be continuously formed even under fully aerobic conditions. LAC from the cytosol enters the mitochondria for further oxidation through intramitochondrial lactate dehydrogenase (mLDH) with the help of the LAC and PYR (pyruvate) transporter present on the outer mitochondrial membrane (identified as monocarboxylic acid transporter 1 [MCT1]), whereas LAC efflux (LAC clearance from cytosol) is carried by MCT4 transporter [1]. In hypoxic conditions, when O_2_ levels are low and anaerobic glycolysis is more prevalent, PYR is primarily reduced to LAC by cytoplasmic lactate dehydrogenase (cLDH) [2,3,4]. The physiological range of tissue [LAC] is 0.5–20 mM [5]. Compared to resting muscles, the cellular LAC levels are elevated in exercising skeletal muscles, and the LAC/ to PYR ratio reaches ≥80 during intense exercise in humans [6,7]. This highlights that the production of LAC exceeds the rate of oxidative metabolism of PYR under these conditions [8]. With elevated LAC levels, there is a reduction in the intracellular pH from pH 6.8–7.2 to pH 5.0–6.5 [9].

Normal LAC levels in serum are 0.6 to 1.8 mM, with hyperlactatemia levels between 2 to 5 mM [10]. If LAC concentrations are >5 mM, the condition is regarded as severe lactic acidosis, and high mortality rates are seen when the LAC concentrations reach >8 mM [11]. Elevated serum LAC concentrations can be seen in acute cardiac patients, reaching ~45 mM (typically with acute coronary syndrome, cardiogenic shock, or cardiac arrest) [12]. Previous studies demonstrated that LAC produced by active muscle is transported to the liver for conversion back to glucose via gluconeogenesis (via the Cori cycle) [13]. Furthermore, recent results have shown that muscle-derived LAC is transported to the heart and serves as an important fuel source [14]. It is also reported that with increasing exercise intensities, there is a transition from fatty acid oxidation (FAO) to an increased use of glucose as a substrate for ATP generation through glycolysis, thus promoting LAC production [15,16]. Whether or not changes in tissue LAC pools due to the rise and fall of tissue LAC production can modify the activities and functions of metabolically important proteins remains to be explored. 

Mb is increasingly appreciated as a metabolite-binding protein with multiple roles. The traditional view of Mb is one of a muscle O_2_-ferrying protein (most abundant in Type I oxidative “red” fibers and cardiomyocytes). Skeletal and cardiac muscle cell mitochondrial O_2_ delivery to the cytochrome c oxidase (COX) involves 2 modes, flow of dissolved O_2_ and Mb-bound O_2_: at least one third of O_2_ uptake, work output, and ATP generation is supported by the latter [17,18,19,20,21]. Substantial evidence in recent years has revealed Mb is not just confined to O_2_ storage and transport but can also be involved in other functions. For instance, Mb (i) is implicated in nitric oxide (NO) scavenging and NO generation [22,23,24,25,26,27], (ii) serves as a fatty acid- and acylcarnitine-binding protein [28,29,30,31,32,33,34,35,36], and (iii) promotes lipid peroxidation [37,38,39,40,41,42]. We have recently proposed that the toggling of oxy- and deoxy-Mb under conditions of changing pO_2_ regulates gene expression and other adaptive activities in part via changes in NO pools, as part of an O_2_-sensing signaling system [43]. Rassaf et al. proposed that deoxy-Mb-derived NO downregulates oxidative phosphorylation, which helps dampen mitochondrial O_2_ consumption when pO_2_ drops [44]. Immunohistochemical and electron microscopy studies showed that Mb and mitochondria colocalize during the exchange of O_2_ [45]. Evidence also suggests that, during the exchange of O_2_ from Mb to mitochondria, oxy-Mb nonspecifically interacts with negatively charged phospholipids of the outer membrane of mitochondria and columbic electrostatics that play a role in its conversion to deoxy-Mb. Furthermore, it was reported that deoxy-Mb more avidly binds to mitochondria than oxy-Mb [46,47,48]. What role, if any, that metabolite binding to Mb plays in modifying these mitochondrial associations is not known. 

A growing body of evidence suggests that, in addition to interactions with gases and lipid metabolites, Mb activities could be impacted by LAC. Previous studies have shown that functional properties of both sperm whale and horse Mb are influenced (allosteric modulation) by high LAC concentrations under acidic conditions [49,50]. It was also shown that, depending on the oxygenation state and at varying pH, Mb may have different affinities toward LAC, behaving as a heterotrophic modulator [50]. However, the stoichiometric binding ratio of Mb to LAC could not be clearly defined through the spectrophotometric method used in those studies. To gather more insight into Mb interactions with LAC and its metabolic consequences, we performed isothermal titration calorimetric (ITC) studies, an O_2_ kinetics evaluation, and time-resolved spectroscopic studies. We hypothesized that the binding of LAC to Mb changes Mb conformation and alters O_2_ binding kinetics in acidic conditions. The current research will help in understanding Mb’s potential role as a LAC shuttle and will help determine if LAC binding influences Mb’s O_2_-sensing and signaling activities or O_2_ delivery to mitochondria.

## 2. Results and Discussion

**Figure 1:**
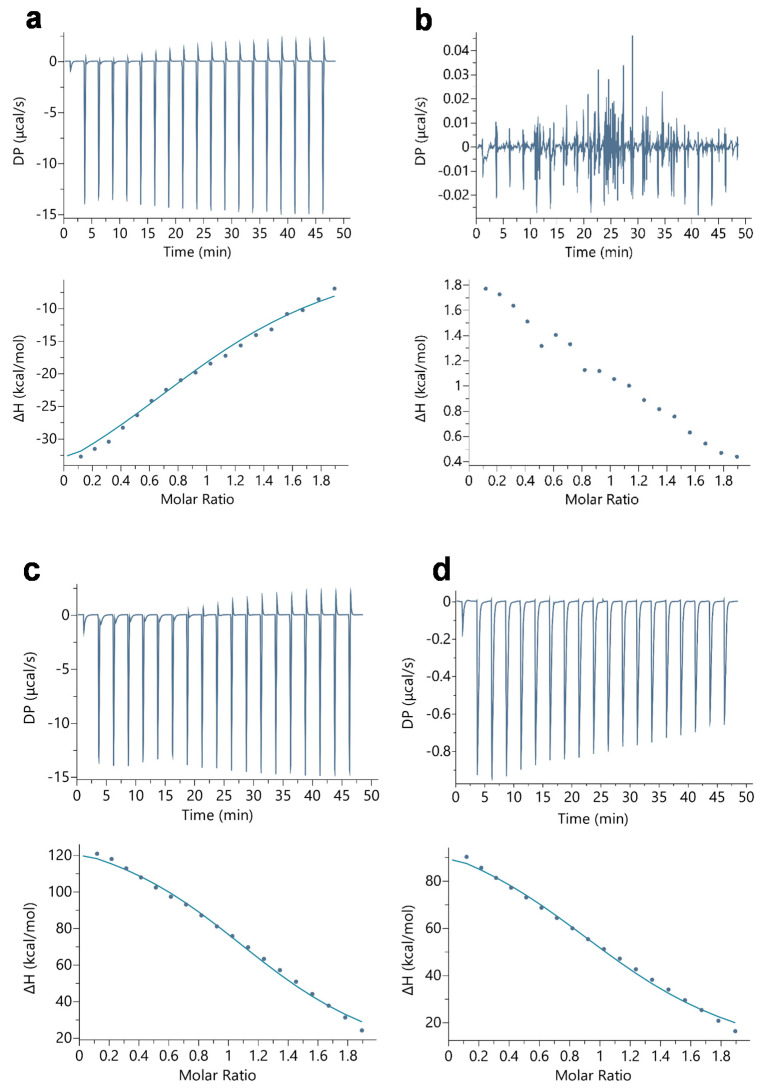

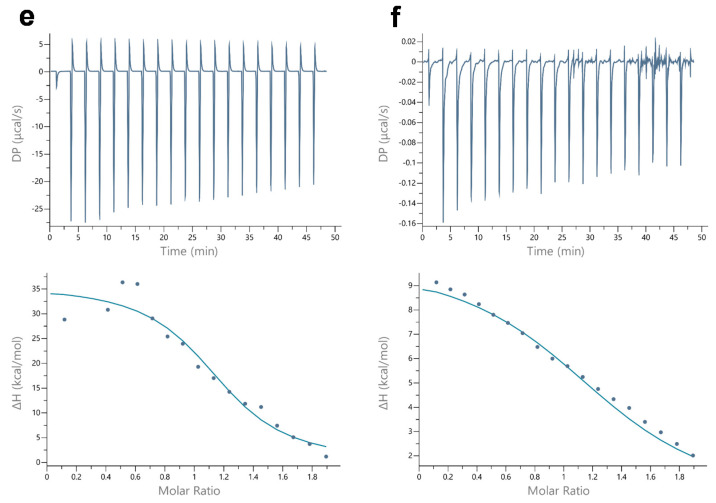
Representative ITC plots of binding of LAC with (**a**) oxy-Mb at pH 7.0, (**b**) deoxy-Mb at pH 7.0, (**c**) oxy-Mb at pH 6.4, (**d**) deoxy-Mb at pH 6.4, (**e**) oxy-Mb at pH 6.0, and (**f**) deoxy-Mb at pH 6.0. Raw data (upper panels) and integrated data (lower panels) represent titration of reactants with time (min) or molar ratios on the x-axis and the energy released or absorbed per injection on the y-axis. The solid lines in the bottom panels represent the best fit of experimental data using a one set of binding sites model provided by the manufacturer’s software (Microcal PEAQ-ITC software). The lower graphs clearly differentiate that at pH 7.0, Mb-LAC binding was predominantly exothermic (downward slope) driven by hydrophilic interactions with large negative enthalpic (∆H) values (details are given in Results and Discussion). In contrast, at acidic pH (pH 6.4 and pH 6.0), the Mb-LAC binding was endothermic (upward slopes) and is mostly favored by hydrophobic interactions and positive ∆H values. All the ITC experiments were repeated 5 times (*n* = 5) to obtain the thermodynamic properties. We have only shown one representative dataset from a single experiment per condition. Statistical analysis was performed with one-tail and two-tail t-test paired two sample for means. All statistical differences in the test data are displayed in superscripts.

Isothermal titration calorimetric (ITC) studies revealed that LAC binds to Mb with equimolar binding stoichiometry, i.e., at a 1:1 molar ratio. Irrespective of a change in pH, the number of binding sites of LAC in Mb was found to be nearly 1 (Table 1). Our results are in agreement with low affinity systems studied through ITC, where *c*-values in the range of 10 ≤ *c* ≤ 500 determine the accuracy of curve fitting to obtain *K_d_* and binding stoichiometry [51]. The ITC binding profiles are shown in Figure 1, where the upper panels display raw binding data of measured potential difference (DP in µcal/s) against run time and the lower panels display the processed data of the change in enthalpy (ΔH in kcal/mol) against molar ratio. Furthermore, the binding experiments showed that at neutral pH (pH 7.0), LAC only bound to oxy-Mb (Figure 1a) and not to deoxy-Mb (Figure 1b). The binding interaction between the oxy-Mb and LAC released heat (i.e., exothermic reaction) with large negative enthalpic (∆H) values (Table 1) favoring the overall binding interaction. The exothermic reaction between Mb and LAC is shown with an upward slope (lower panel of Figure 1a). Additionally, signature plot analysis clearly indicates that the binding interaction between oxyMb and LAC at pH 7.0 is predominantly driven by the change in enthalpy (∆H) (Figure S1a). This interaction likely represents hydrophilic interactions that play a major role in stabilizing the Mb+LAC complex. Moreover, a negative ΔG value of −6.2 kcal/mol determined the overall stability of the protein-ligand complex. On the other hand, LAC avidly bound to both oxy- and deoxy-Mb at acidic pH (pH 6.4 and pH 6.0) (Figure 1c–f). Additionally, the binding interaction between Mb and LAC in acidic pH conditions is endothermic, resulting in a downward slope pattern shown in lower panels of Figure 1c–f. The Mb-LAC binding was mostly favored by a change in entropy (ΔS) (Figure S1b–e), primarily representing hydrophobic interactions in stabilizing the Mb+LAC complex. A more negative ΔG value of −6.9 kcal/mol for the oxy-Mb+LAC complex at pH 6.4 and −7.8 kcal/mol for the Mb+LAC complex at pH 6.0 indicated that, these complexes were highly stable when compared to the Mb+LAC complex at pH 7.0 (Table 1). Similarly, the ΔG value for the Mb+LAC complex at pH 6.0 was more negative than for the Mb+LAC complex at pH 6.4, except at pH 7.0 where no binding was observed (Figure S1). Therefore, based on ΔG values, the stability of the Mb+LAC complex(es) are in the order of oxy-Mb+LAC (pH 6.0) > deoxy-Mb+LAC (pH 6.0) > oxy-Mb+LAC (pH 6.4) > deoxy-Mb+LAC (pH 6.4) > oxy-Mb+LAC (pH 7.0). This further confirms that binding LAC to Mb is comparatively more stable at acidic conditions. Studies by Sankaranarayanan [52] revealed that the decrease in buffer pH from pH 7.0 to pH 6.4 marginally changes the secondary structure of Mb with a decrease in the % α-helix (100% at pH 7.0 to 97.2% at pH 6.5). Additional computational and X-ray crystallography studies may warrant detailed insights on the structural and conformational changes in the secondary and tertiary structure of Mb protein with LAC binding in different pH conditions.

**Figure 2:**
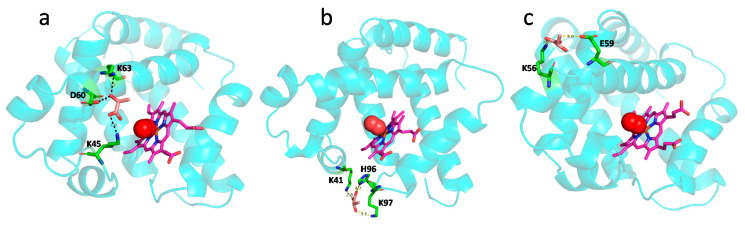
Autodock results displaying LAC interaction with oxy-Mb residues at (**a**) pH 7.0, (**b**) pH 6.4, and (**c**) pH 6.0. At pH 7.0, LAC shows interaction with residues K45, K63, and D60, while at pH 6.4, LAC shows interaction with residues K41, H96, and K97, and at pH 6.0, LAC shows interaction with residues K56 and E59. LAC (brown), heme center (pink), and the residues (green) interacting with LAC are displayed as sticks. Mb protein (cyan) is displayed as ribbon structure an oxygen (red) in spheres. Possible hydrogen bond interactions between side chains of residues and LAC are displayed as dashed yellow lines with bond length.

**Figure 3:**
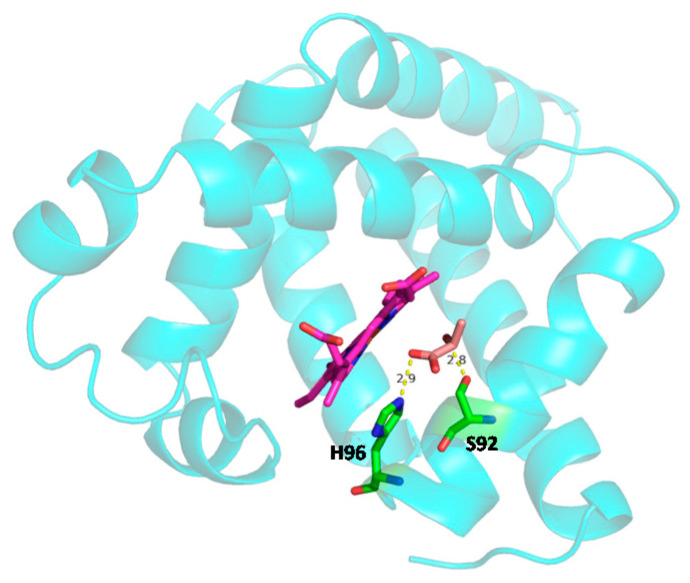
Autodock results displaying LAC interaction with deoxy-Mb residues. Irrespective of the change in acidic pH (pH 6.4 and pH 6.0), LAC is docked near to proximal His side of heme center of deoxy-Mb, interacting with the residues H96 and S92, except at pH 7.0, where no LAC binding was observed. LAC (brown), heme center (pink), and the residues (green) interacting with LAC are displayed as sticks. Mb protein (cyan) is displayed as ribbon structure. Possible hydrogen bond interactions between side chains of residues and LAC are displayed as dashed yellow lines with bond length.

ITC studies also revealed the binding affinity (*K_a_*, inverse of *K_d_*) of LAC interaction with oxy- and deoxy-Mb in different pH conditions. Specifically, with the drop in pH from pH 7.0 to pH 6.0, the *K_a_* of LAC for oxy-Mb was increased by ~3-fold, and for deoxy-Mb at pH 6.4, the *K_a_* was ~2-fold higher than pH 6.0 (Table 1). The plausible reason might be due to a change in protonation states of Mb residues (in particular, histidine, His) in acidic pH conditions. Computational analysis revealed that Mb net charge (p*K_a_*) was increased with decreasing pH, i.e., p*K_a_* of ~2.6, ~6.3, and ~8.9 at pH 7.0, pH 6.4, and pH 6.0, respectively. The protonation states of His residues in Mb were also increased with decreasing pH. At pH 7.0, no His residues were protonated, since the isoelectric pH (p*I*) of Mb was ~7.8. However, at pH 6.4, His36 was protonated and at pH 6.0, His residues at positions 36, 48, 81, 82, 97, and 226 were found to be protonated (Figure S2). Apart from the protonation sites, another compelling reason might be the change in the binding site of LAC during interaction with Mb in different pH conditions. It was also reported that Mb does not contain any specific binding location for LAC and LAC may act as an allosteric regulator [50]. Molecular docking studies revealed that LAC putatively binds to oxy-Mb at different locations with respect to changes in pH. Nevertheless, LAC binds near the distal His side of the heme center via hydrogen bonding interactions in all conditions, except for oxy-Mb at pH 6.0 (Figure 2). In oxy-Mb, at pH 7.0, LAC is docked near the oxygen binding site (proximal His residue) of the heme center, interacting with the residues K45, D60, and K63 (Figure 2a). However, with the decrease in pH, LAC binds to oxy-Mb away from the heme binding site. At pH 6.4, LAC interacts with residues K41 and K97 near the heme binding site (Figure 2b), whereas at pH 6.0 LAC interacts with residues K56 and E59 away from heme binding center (Figure 2c). Irrespective of pH, LAC is docked near to proximal His side of the heme center of deoxy-Mb interacting with the residues H96 and S92 (Figure 3). No detectable binding of LAC with deoxy-Mb was observed at pH 7.0. Future studies involving molecular dynamics simulation and quantum mechanics will help in calculating the stability and binding energies of LAC interaction with oxy- and deoxy-Mb in various pH conditions to reveal more insights into the structural and conformational changes of all the Mb+LAC complex(es). However, the present ITC binding results and molecular docking predictions strengthen our hypotheses related to different affinities of oxy-Mb toward LAC in varied pH conditions. In parallel, ITC binding experiments were also validated with a control protein, LYZ, that showed no detectable binding with LAC in all the tested pH conditions (Figure S3a–c). Our results contrast with the earlier studies by Giardina et al., in which binding studies were performed at pH 6.5 and showed that LAC had a lower affinity for oxy-Mb than deoxy-Mb (*K_d_* values: 26 × 10^3^ µM vs. 2.5 × 10^3^ µM, respectively) [50]. However, we found that LAC showed a higher affinity toward oxy-Mb compared to deoxy-Mb at pH 6.4 (~7 µM vs. ~14 µM, respectively; Table 1). One of the reasons for different results between the two studies might be differences in measuring thermodynamic binding. The earlier binding studies were determined spectrophotometrically using tonometry, whereas our binding kinetic studies were performed using an advanced ITC method that precisely reflects protein-ligand stoichiometry. Importantly, both studies concur in identifying LAC as a binding metabolite for Mb.

**Figure 4:**
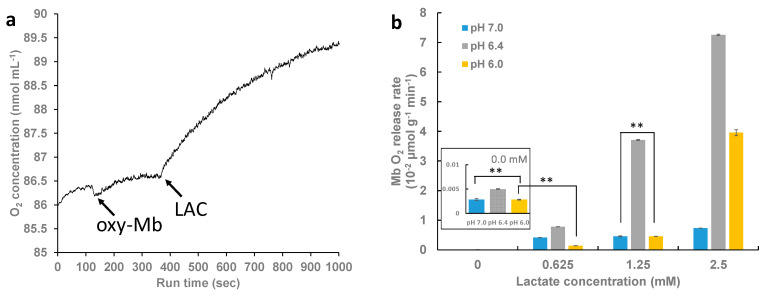
Effect of lactate binding to oxy-Mb and O_2_ release kinetics. Oxygen kinetics is studied using Oxytherm+ respirometer. All experiments were performed with 50 mM sodium phosphate buffer (pH 7.0, pH 6.4, and pH 6.0) containing 150 µM equine oxy-Mb and varying concentrations of lactate (0.625 mM to 2.5 mM) and oxy-Mb alone as a control. (**a**) A representative graph showing addition of lactate eliciting a rapid release of O_2_ from oxy-Mb at pH 6.4. Arrows in the figure indicate the point of addition of oxy-Mb and LAC into buffer during the run. (**b**) Rate of release of O_2_ from Mb against lactate concentrations with buffers (pH 7.0, pH 6.4, and pH 6.0). Inlet chart displays rate of release of O_2_ from deoxy-Mb alone (pre-LAC) conditions. All O_2_ kinetic experiments were repeated 3 times (*n* = 3). Statistical analysis was performed using one-way ANOVA and the Tukey–Kramer post hoc test. Statistical analysis revealed no significant difference in O_2_ release from oxy-Mb alone (without LAC) at pH 7.0 and pH 6.0. Additionally, no significant difference in O_2_ release was observed at pH 7.0 and pH 6.0 with 1.25 mM LAC condition. Moreover, with the addition of 0.625 mM LAC to oxy-Mb at pH 6.0, no significance difference in O_2_ release was observed when compared to oxy-Mb alone at pH 6.0. Among all the test groups, only statistically not significant groups are shown with horizontal lines and two asterisks.

**Figure 5:**
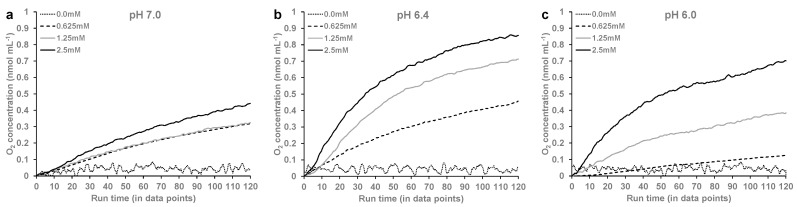
The O_2_ release at varying concentrations (0.0 mM, 0.625 mM, 1.25 mM, and 2.5 mM) of LAC at (**a**) pH 7.0, (**b**) pH 6.4, and (**c**) pH 6.0. The rate of O_2_ release was calculated from the linear portion of the graphs immediately after addition of LAC to oxy-Mb. Representative data from a single experiment per condition are shown here. All O_2_ kinetic experiments were repeated 3 times (*n* = 3).

O_2_ kinetic studies in sodium phosphate buffer in different pH conditions with the addition of different concentrations of LAC showed that the addition of LAC to oxy-Mb resulted in the release of O_2_. A representative graph depicting the rapid release of O_2_ after the addition of LAC to the oxy-Mb solution at pH 6.4 is shown in Figure 4a. Representative graphs showing the release of O_2_ from oxy-Mb with the addition of LAC and without LAC in different pH conditions are shown in Figure 5a–c. With an increasing incubation time of LAC with oxy-Mb, the rate of O_2_ release from oxy-Mb was decreased; thus, the rate of O_2_ release was obtained from the linear portion of the graphs immediately after the addition of LAC to oxy-Mb. Irrespective of pH, with oxy-Mb solution alone (pre-LAC), little to no change in the O_2_ levels were observed (inset of Figure 4b and Figure 5a–c). This confirms that O_2_ release from oxy-Mb is only due to the interaction of LAC with oxy-Mb. At more acidic pH levels, rising LAC concentrations increased the rate of release of O_2_ from oxy-Mb (see grey and yellow bars, Figure 4b), but at pH 7.0, increasing LAC had a marginal effect (see blue bars, Figure 4b). A maximum rate of O_2_ release (72 nmol/min/g protein) from oxy-Mb was observed with 2.5 mM LAC in pH 6.4 buffer. Additionally, our data is supported by the O_2_ release rate constants (mM LAC/nM O_2_ release) of 0.04, 0.029, and 0.537 at pH 6.0, pH 6.4, and pH 7.0, respectively. This further strengths our results on maximum O_2_ release observed with lower concentrations of LAC at acidic conditions when compared to physiological pH. The complex nature of pH-dependent changes in O_2_ release from oxy-Mb might be due to (i) changes in the protonation state(s) of Mb with respect to pH or (ii) LAC binding near the O_2_ exit pathway hindering O_2_ release. These open questions need further comprehensive investigation. Based on the results, we hypothesize that the abundance of LAC in cells could impinge upon the association of O_2_ to Mb, especially as pH drops. This, in turn, could impact the availability of O_2_ from oxy-Mb to support mitochondrial bioenergetics or could alter other Mb activities that depend on the oxygenation state of the protein (e.g., its putative role as an O_2_-sensor). Considering the emerging role of oxy-Mb as a fatty acid- and long-chain acylcarnitine-binding protein [28,29,30], it is intriguing to speculate that the LAC-induced release of O_2_ from oxy-Mb could mean that conditions of lower O_2_ and increased LAC would decrease the binding of lipids that coincide with a transition to deoxy-Mb. If true, this may play a physiological role in terms of regulating fatty acid availability for oxidative and non-oxidative fates. In addition, under this working model, lipotoxicity associated with muscle or myocardial ischemia may partly stem from excessive unbound (free) intracellular pools of FAs and acylcarnitines due to off-loading from Mb as it transitions to deoxy-Mb, an idea we have considered previously [28,29,30].

**Figure 6:**
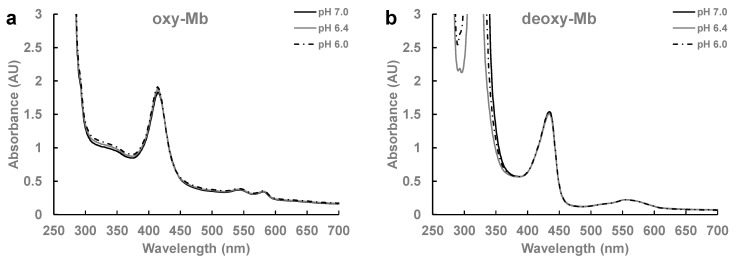
A representative UV-Vis spectra of freshly prepared (**a**) oxy-Mb and (**b**) deoxy-Mb samples each at pH 7.0, pH 6.4, and pH 6.0. Preparation of protein samples is detailed in the Materials and Methods section. All the spectra were repeated 3 times (*n* = 3).

Freshly prepared oxy-Mb preparations showed the expected absorption maxima at 418 nm, and the *β* and *α* bands at 548 and 579 nm, respectively (Figure 6a). Similarly, deoxy-Mb preparations showed the expected absorption maxima at 435 nm and a single broad peak at 557–562 nm (Figure 6b). This confirms that our enriched Mb preparations were uniform without any partial mixture. However, it was reported that complete removal of dissolved O_2_ from any liquid solution is typically not achievable by purging N_2_ gas into liquid solutions even for prolonged time periods, and that the presence of minute concentrations of dissolved O_2_ (<0.1–0.3 ppm) in the N_2_ purged liquid solutions cannot be ruled out [53]. If any minor amount of dissolved O_2_ is present in our protein solution preparation, our time-resolved spectroscopy studies showed no change in Mb heme conformation even after prolonged time periods (up to 45 min), similar to the freshly prepared Mb solution as shown in Figure 6a. This confirms that the minor amount of dissolved O_2_ in our Mb preparations had no interference on all our biochemical experiments, i.e., ITC and O_2_ kinetics, since the time required to complete each ITC binding experiment was <45 min and the O_2_ kinetic experiment was <15 min. Similarly, no change in spectral data were observed with LAC-incubated oxy-Mb in all the test pH conditions. However, marginal changes in the heme peak absorbance (435 nm) with a significant decrease in peak absorbance around 300–315 nm (not known) were observed with LAC-incubated deoxy-Mb protein, which may be attributed to ligand-induced conformational changes of protein (data not shown). Further time-resolved circular dichroism studies are warranted to determine detailed structural insights of LAC bound Mb.

Since the LAC binding site of Mb has not been established, several authors have studied the crystal structures of Mb derivatives and predicted possible hydrogen bonding interactions between the anion binding site of Mb with His E7, Arg CD3, and Thr E10 residues (catalytic triad) [54]. A distal histidine group (His E7) placed on the opposite side of the heme also serves as a small molecule binding site (vacant in deoxy-Mb) that holds gaseous ligands such O_2_, NO, and CO [26,27,54,55,56,57]. Experiments have shown that His-E7 acts as a gate for small molecule access (moving in and out) for the active site even under low pH (pH 4.6–6.5) conditions [58,59,60,61,62,63]. Depending on the open or closed conformation, the entrance of the small apolar diatomic ligands is only possible with the minor rotation of the imidazole side chain of the distal His-E7 channel. Experiments on the crystal structure of sperm whale oxy-Mb incubated with LAC at pH 6.5 do not show a specific LAC-binding pocket [50]. Further investigations using docking studies and molecular dynamics simulations will provide more insight into the possible specific binding sites of LAC near the heme pocket of Mb, which, if present, could impact O_2_ release with increasing cellular LAC levels and concomitant interconversion of oxygenated forms to deoxygenated forms of Mb. 

## 3. Materials & Methods

### 3.1. Materials

Horse heart muscle myoglobin (Mb), chicken egg white lysozyme, sodium lactate (LAC), and sodium dithionite were purchased from Sigma Aldrich, St. Louis, MO, USA. All other chemicals used in the experiments are of analytical grade and were also procured from Sigma Aldrich, St. Louis, MO, USA.

### 3.2. Preparation of Mb

Preparations enriched in oxygenated and deoxygenated forms of Mb were prepared as described by our earlier publication related to Mb interaction with fatty acids and acylcarnitines [30]. Briefly, 500 µM of Mb was dissolved in 50 mM sodium phosphate buffer of desired test pH. To promote conversion of ferric (Fe^3+^) to ferrous (Fe^2+^) iron, 3 mM of sodium dithionite was added to the protein solution with gentle mixing. Thereafter, the solution was subjected to a desalting column to remove the reducing agent and to remove the interference, particularly in O_2_ release kinetics and UV-Vis spectroscopy absorbance peaks. However, ligand binding studies using a Microcal instrument (described later) were performed in the presence of sodium dithionite, as it did not show any effect on binding properties. Thereafter, purging the protein solution enriched in oxy- and deoxy-Mb with either O_2_ or N_2_ gas, respectively, was performed continuously for 10 min. Formation of oxy- and deoxy-Mb were confirmed based on their characteristic peaks using UV-visible spectroscopy. All the experiments were performed in three different pH conditions (7.0, 6.4, and 6.0), mimicking intracellular physiological and acidic pH states that would be typically observed in skeletal muscle cells in “rested” and “active” (hyperlactatemia) conditions.

### 3.3. Ligand Binding Studies

Protein-ligand binding experiments were performed using isothermal titration calorimetry (ITC) (Microcal PEAQ-ITC, Malvern, Chester County, PA, USA). Before starting the experiment, both the ligand solution and the protein solution were purged with either O_2_ or N_2_ for 10 min. Both the protein and LAC solutions were thermally equilibrated to 25 °C prior to the start of the titration. LAC was loaded in the reaction cell at an initial concentration of 50 µM and titrated against 500 µM of either oxy- or deoxy-Mb solution, maintaining a 1:10 ratio between ligand and protein. To obtain the working *c*-value between 10 ≤ *c* ≤ 500 [51], different protein to ligand molar ratios (1:2, 1:5, 1:10, and 1:20) were tested, and based on the *c*-value, the molar ratio of 1:10 was selected for all ITC binding studies. A total of 19 injections (2 µL each) from the syringe were used to generate the ITC curves within each experiment. During the experimental run, the samples were mixed thoroughly at a constant stirring rate of 750 rpm. Between each injection, a 150 s gap was maintained to achieve a stable baseline. Data obtained from the ITC experiments were best fit to a one set of binding sites model provided by Microcal PEAQ-ITC software (version 1.40). Heats of dilution and heats caused by potential products formed during the course of the ITC experiments were corrected by performing appropriate blank titrations, consisting of (a) either oxy- or deoxy-Mb in the test buffer solution, (b) test buffer in the LAC, and (c) buffer-buffer solution. Lysozyme was used as a negative control protein in the protein-ligand binding experiments. All the binding experiments were performed 5 times (*n* = 5) and data obtained from statistical analysis are presented here. 

The change in entropy (ΔS) was calculated using the equation:

ΔG = ΔH − TΔS(1)

where ΔG represents the change in Gibbs free energy, ΔH is the change in enthalpy, and T is the absolute temperature. 

*c* values were calculated using the equation:

*c* = *nK_a_*[M]_t_ (2)

where *n* is the number of binding sites per receptor (macromolecule), [M]_t_ is macromolecule concentration, and *K_a_* is the association constant.

### 3.4. Oxygen Kinetic Studies

The O_2_ concentrations (release and binding) during the ligand interactions with Mb in the solution were measured using Oxytherm+ liquid-phase oxygen electrode system (Hansatech Instruments, Norfolk, UK). The Oxytherm+ respirometer is an advanced instrument routinely used for respiration studies.

The measurement of dissolved O_2_ is calculated at the given temperature and atmospheric pressure according to the following equation [64]:

Cs = 14.16 − (0.394 × T) + (0.007714 × T^2^) − (0.0000646 × T^2^) (3)

where Cs is the saturated O_2_ concentration in ppm and T is the temperature in °C.

1 ppm is equivalent to 1 µg/mL or (1 µg/32 g/mol) = 0.03125 µmol/mL or 31.25 nmol/mL.

The optimum concentration of oxy-Mb was found to be 25 µM, and LAC concentrations were varied from 620 µM to 50 mM. Similarly, deoxy-Mb protein alone (i.e., without LAC) was also tested. All the experiments were carried out at a constant temperature of 25 °C using a Peltier thermostat. The solutions were mixed at 50 rpm using a small magnetic stirring bar placed inside the sample container. Samples were injected into the buffer solution (50 mM sodium phosphate) at varying pH levels (pH 6.0–7.0) using a Hamilton glass syringe (1*cc*) after achieving equilibrium. Ligand was added to the Mb protein. Appropriate buffer controls voiding either Mb or LAC were used to nullify any artefacts. O_2_ kinetic experiments were also performed. All the kinetic experiments were performed 3 times (*n* = 3) and data obtained from statistical analysis are presented here.

### 3.5. Time-Resolved Spectroscopic Studies

The change in the spectral characteristics of Mb alone and LAC-bound Mb were recorded using UV-Vis spectroscopy (Polarstar Omega, BMG Labtech, ortenberg, Germany). All the sample preparations and the concentrations used were similar to the ITC experiments. After degassing Mb and LAC samples either with O_2_ or N_2_ gas in sealed vials, appropriate volumes of the samples were collected using a Hamilton glass syringe (1*cc*), mixed in a glass cuvette, and sealed immediately; time-resolved spectra were recorded immediately at 25 °C for a period of 75 min. All spectral data were collected at 5 min time intervals. All the spectral experiments were performed 3 times (*n =* 3). 

### 3.6. Molecular Docking

Autodock 4.2 [65] was used to dock LAC to the heme binding pocket of deoxy-Mb (PDB: 2V1K). We used the relaxed model of the oxy-Mb structure derived from horse deoxy-Mb (due to the unavailability of the oxy-Mb crystal structure), which is used in our previous studies [29]. A three-dimensional structure of LAC was obtained from PubChem database (https://pubchem.ncbi.nlm.nih.gov/compound/Lactate, accessed on 23 April 2022). The iron ion parameters in the heme group were obtained from Autodoc 4.2 software. The iron ion was selected directly from the Set Map Types within the Grid tab to add iron with default parameters for docking. The protonation state of each titratable residue in Mb at different pH values was set based on pKa estimations by PROPKA [66]. AutoDock Tool was then used to prepare the protein-ligand system by assigning polar hydrogen atoms and Kollman’s partial charges with solvation parameters to the protein. A grid box of search space (70 Å × 70 Å × 70 Å) enclosing the heme group and residues within 5 Å from the heme center with a grid spacing of 0.375 Å was assigned. A Lamarckian genetic algorithm (LGA) was applied with a population size of 300 and 25 million maximum energy evaluations for 150 independent runs. The best docking structure is selected based on the lowest binding energy within the largest cluster of the docking results.

### 3.7. Statistical Analysis

Statistical analysis was performed using Microcal Origin software via an iterative algorithm for all ITC binding experiments. Similarly, nonlinear regression analysis of the average data points was calculated for each condition. One-way ANOVA was performed to determine the statistically significant differences between the data obtained from the binding studies in different pH conditions with a level of confidence of 95%. Additionally, one-tail and two-tail t-test paired two sample for means was performed. For oxygen kinetic analysis, both one way ANOVA and the Tukey–Kramer post hoc test were performed to determine the statistical significance. All the experimental results data are presented as means ± standard error (SE).

## 4. Conclusions

The biochemical results presented herein are consistent with a proposed model in which LAC binding to Mb is a dynamic process within Mb-expressing tissues, regulated by a combination of factors including pH, pO_2_, Mb oxygenation status, and metabolite concentration. Under this model, when LAC concentration is low at neutral pH (pH 7.0), as would be seen in resting muscle, LAC does not interact with deoxy-Mb but is bound to oxy-Mb. However, as the cell slips into acidic conditions and lower pO_2_ during high energy demand, increased LAC levels would drive interactions with both oxy- and deoxy-Mb and promote the off-loading of O_2_ from oxy-Mb. If true, this off-loading may be an adaptational response to lowered pO_2_, in that Mb-bound O_2_ becomes more available for metabolic processes. LAC binding also may bring a conformational change in the Mb protein, as suggested by spectral changes in the ligand-bound state, and this may influence Mb functions beyond the provision of O_2_ in support of oxidative phosphorylation. For instance, based on phenotype and gene expression patterns of Mb knockout mice [43,67], we proposed that an important role for Mb is an O_2_ “sensor” that regulates metabolic and gene expression pathways through mechanisms responsive to toggling between oxy- and deoxy-Mb [43]. It is intriguing to consider that such processes could be impacted by small molecule metabolite binding, including lipid derivatives [28,29,30] and LAC. Furthermore, we speculate that Mb-LAC interactions play a pivotal role in the transport of LAC by deoxy-Mb into the mitochondrial membrane space and ultimately into the matrix. In other words, Mb may serve as a transporter that aids intracellular LAC shuttling into the mitochondria. How changes in Mb and cellular pH influence mitochondrial function and metabolism remains to be fully determined, but there is now a growing body of evidence to suggest that Mb-LAC interactions could play an important role in metabolic regulation. 

## Figures and Tables

**Table 1:** Thermodynamic binding properties of lactate titrated against equine deoxy-Mb and oxy-Mb in ITC experiments in different pH conditions. Dissociation constant (*K*_d_), association constant (*K*_a_), the ratio of the receptor concentration, and the dissociation constant are determined by *c* value. Change in enthalpy (ΔH°) and change in entropy (ΔS°) are shown in the table. All the ITC experiments were repeated 5 times (*n* = 5) to obtain the thermodynamic properties. Statistical analysis was performed using one-way ANOVA and both one-tail and two-tail *t*-test paired two sample for means for each parameter, separately between oxy-Mb and deoxy-Mb at each pH condition.

Thermal Properties	pH 7.0		pH 6.4		pH 6.0	
	oxy-Mb	deoxy-Mb	oxy-Mb	deoxy-Mb	oxy-Mb	deoxy-Mb
K_d_ (µM)	20.7 ± 2.7 ^a^*	-	6.9± 1.1 ^b1^	13.7± 1.6 ^c1^*	1.9 ± 0.2 ^d2^	6.4 ± 0.2 ^b1^
K_a_ (µM)	0.05 ± 0.0 ^a^*	-	0.2 ± 0.03 ^b1^	0.1± 0.0 ^c1^*	0.5 ± 0.04 ^d2^	0.15 ± 0.0 ^b1^
*c* value	22.4 ± 1.4 ^a^	-	67.5 ± 15.5 ^b1^	30.4 ± 4.6 ^c1^*	267.9 ± 30.4 ^d2^	61.2 ± 0.9 ^b1^
ΔH° (kcal mol^−1^)	−53.1 ± 0.3 ^a^*	-	151.5 ± 18.5 ^b1^	149.5 ± 13.0 ^b1^	34 ± 3.5 ^c2^	12.6 ± 0.4 ^d3^
ΔS° (cal mol^−1^ K^−1^)	−0.15 ± 0.01 ^a^*	-	0.5 ± 0.06 ^b1^	0.55 ± 0.03 ^b1^	0.14 ± 0.01 ^c2^	0.1 ± 0.0 ^d3^
No. of binding sites	0.9 ± 0.7 ^b^*	NDB	0.8 ± 0.03 ^a1^	0.8 ± 0.03 ^a1^	1.0 ± 0.06 ^a2^	0.8 ± 0.02 ^a2^

NDB: no detectable binding; superscript displays the statistical differences among the experimental results; lower-case alphabets in superscript denotes one-tail *t*-test and numerical values and asterisk symbol in superscript denotes two-tail *t*-test.

## Data Availability

Not Applicable.

## Supplementary Materials

The following supporting information can be downloaded at: https://www.mdpi.com/article/10.3390/ijms23094747/s1.

## Abbreviations

deoxy-Mb	deoxygenated myoglobin
LAC	sodium lactate
Mb	myoglobin
oxy-Mb	oxygenated myoglobin
