# Supplementary material for: Myoglobin Interaction with Lactate Rapidly Releases Oxygen: Studies on Binding Thermodynamics, Spectroscopy, and Oxygen Kinetics
Source: Int J Mol Sci. 2022 Apr 26;23(9):4747. doi: 10.3390/ijms23094747 (PMC9103699; doi:10.3390/ijms23094747)
Supplement: Supplementary file 1 [file ijms-23-04747-s001.zip › ijms-1678837-supplementary.pdf]

International Journal of
*Molecular Sciences*

MDPI

*Article*

# Myoglobin Interaction with Lactate Rapidly Releases Oxygen: Studies on Binding Thermodynamics, Spectroscopic and Oxygen Kinetics

**Kiran Kumar Adepu [1,2,*], Dipendra Bhandari [1], Andriy Anishkin [3], Sean H. Adams [4,5] and Sree V. Chintapalli [1,2,*]**

[1] Arkansas Children's Nutrition Center, Little Rock, AR 72202, USA; bhandarid@archildrens.org
[2] Department of Pediatrics, University of Arkansas for Medical Sciences, Little Rock, AR 72202, USA
[3] Department of Biology, University of Maryland, College Park, MD 20742, USA; anisan@gmail.com
[4] Department of Surgery, School of Medicine, University of California, Davis, CA 95616, USA; shadams@ucdavis.edu
[5] Center for Alimentary and Metabolic Science, University of California, Davis, CA 95616, USA
* Correspondence: kkadepu@uams.edu (K.K.A.); svchintapalli@uams.edu (S.V.C.)

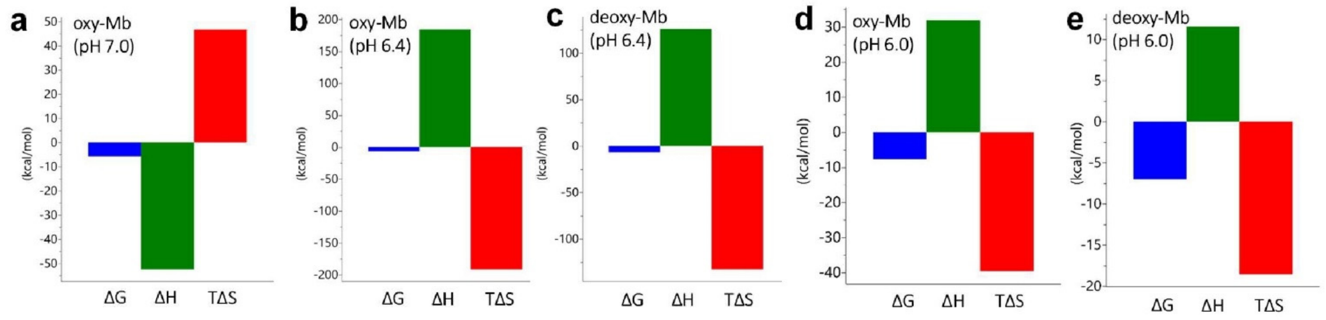


**Supplementary Figure S1.** A representative ITC signature plot of oxy-Mb bound lactate at pH 7.0 (deoxy-Mb did not bind LAC at pH 7.0) (a), oxy-Mb bound LAC (b) and deoxy-Mb bound LAC (c) at pH 6.4, oxy-Mb bound LAC (d) and deoxy-Mb bound LAC (e) at pH 6.0.

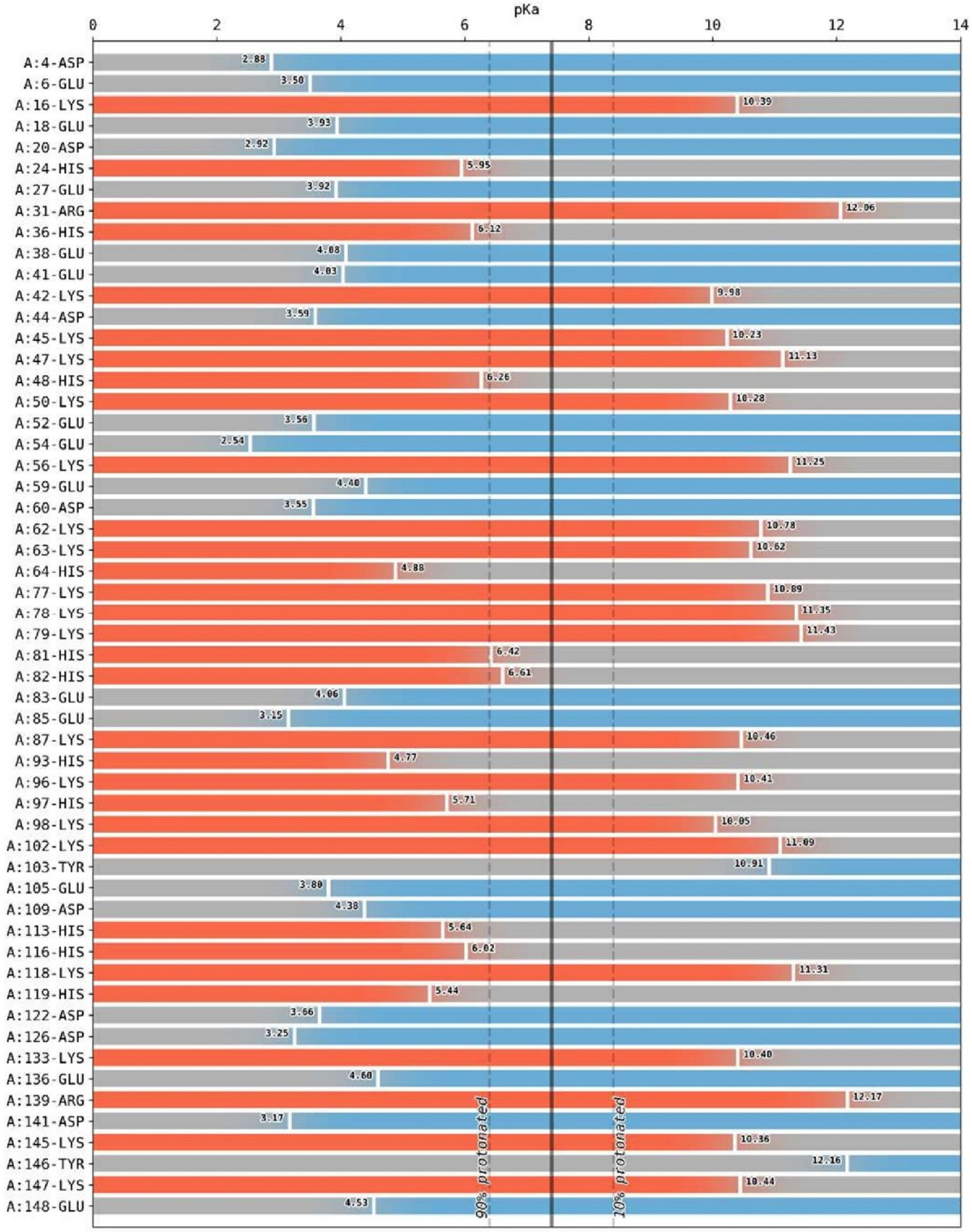


**Supplementary Figure S2:** Different pKa values of residues of horse heart muscle Mb (PDB ID: 2V1K) determined by PROPKA.

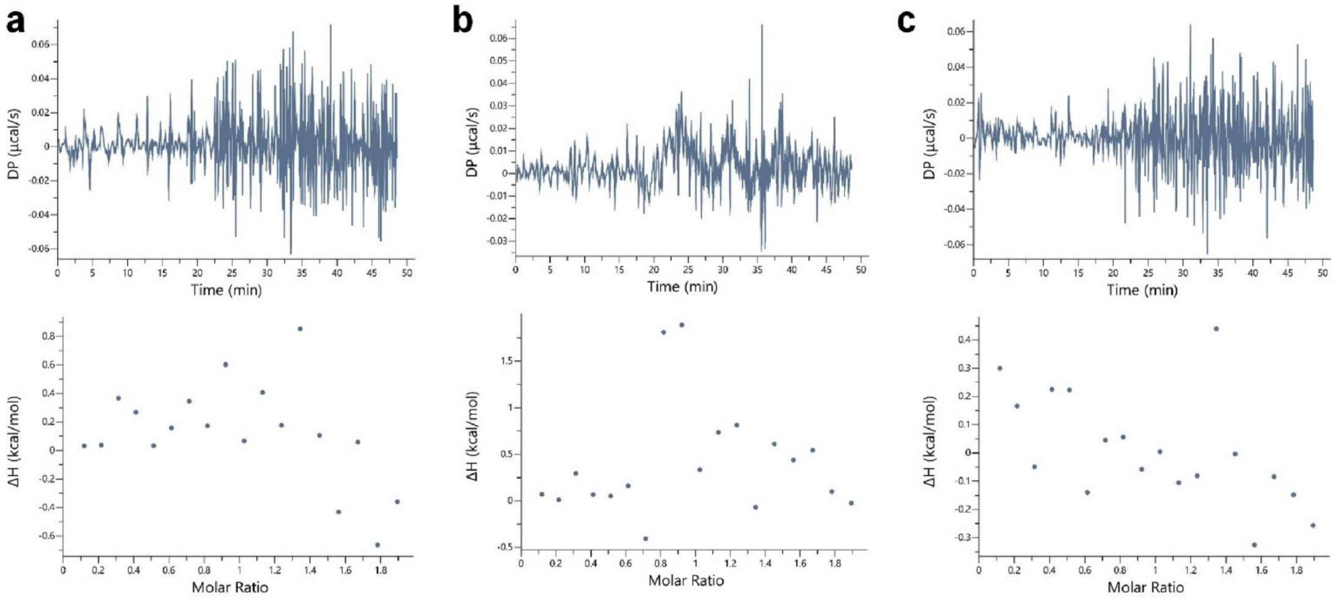


**Supplementary Figure S3.** Representative ITC plots of binding interactions of LAC with lysozyme at **(a)** pH 7.0, **(b)** pH 6.4 and **(c)** pH 6.0. LAC did not bound to lysozyme in all the test pH buffers. Thus, lysozyme was treated as a negative control protein in all the ITC binding studies.
